# Structural basis of human TREX1 DNA degradation and autoimmune disease

**DOI:** 10.1038/s41467-022-32055-z

**Published:** 2022-07-25

**Authors:** Wen Zhou, Desmond Richmond-Buccola, Qiannan Wang, Philip J. Kranzusch

**Affiliations:** 1 Department of Immunology and Microbiology, School of Life Sciences, Southern University of Science and Technology, Shenzhen, Guangdong 518055 China; 2 Department of Microbiology, Harvard Medical School, Boston, MA 02115 USA; 3 Department of Cancer Immunology and Virology, Dana-Farber Cancer Institute, Boston, MA 02115 USA; 4 Parker Institute for Cancer Immunotherapy at Dana-Farber Cancer Institute, Boston, MA 02115 USA

**Keywords:** Innate immunity, X-ray crystallography

## Abstract

TREX1 is a cytosolic DNA nuclease essential for regulation of cGAS-STING immune signaling. Existing structures of mouse TREX1 establish a mechanism of DNA degradation and provide a key model to explain autoimmune disease, but these structures incompletely explain human disease-associated mutations and have limited ability to guide development of small-molecule therapeutics. Here we determine crystal structures of human TREX1 in apo and DNA-bound conformations that provide high-resolution detail of all human-specific features. A 1.25 Å structure of human TREX1 establishes a complete model of solvation of the exonuclease active site and a 2.2 Å structure of the human TREX1–DNA complex enables identification of specific substitutions involved in DNA recognition. We map each TREX1 mutation associated with autoimmune disease and establish distinct categories of substitutions predicted to impact enzymatic function, protein stability, and interaction with cGAS-DNA liquid droplets. Our results explain how human-specific substitutions regulate TREX1 function and provide a foundation for structure-guided design of TREX1 therapeutics.

## Abstract (web summary)

TREX1 is a cytosolic DNA nuclease and the genetic mutations of it are linked to autoimmune diseases. Here the authors determine the first structure of the human TREX1–DNA complex, and provide a new foundation to explain how patient TREX1 mutations cause autoimmune disease.

## Introduction

Cytosolic DNA sensing enables innate immune recognition of pathogen replication and DNA damage. However, the immune response to DNA must be precisely regulated to maintain immune homeostasis and prevent autoimmunity^1^. In human cells, a major sensor of cytosolic DNA is the enzyme cyclic GMP–AMP synthase (cGAS) that directly binds DNA and catalyzes the synthesis of the signaling molecule 2′–5′, 3′–5′ cyclic GMP–AMP (2′3′-cGAMP)^2–6^. 2′3′-cGAMP functions as a second messenger that directly binds the receptor Stimulator of Interferon Genes (STING) to induce immune responses via type I interferon and NF-κB signaling. Inappropriate activation of cGAS-STING immunity is directly linked to autoimmune disorders, including Aicardi-Goutières Syndrome, familial chilblains lupus, and retinal vasculopathy with cerebral leukodystrophy^7,8^. Frequently, these autoimmune diseases are the result of mutations that inactivate a negative-regulator of cGAS-STING signaling named three-prime repair exonuclease 1 (TREX1). Loss of TREX1 function results in accumulation of cytosolic DNA, inappropriate activation of cGAS-STING signaling, and severe autoimmunity^9–13^. Additionally, TREX1 degradation of tumor-derived DNA has been implicated in limiting antitumor immunity and cancer immunotherapies^14–18^. The clear role of DNA degradation in controlling cytosolic DNA sensing demonstrates the importance of understanding the biochemical and structural basis of human TREX1 function.

Human TREX1 is a 3′-to-5′ exonuclease composed of an N-terminal DNase catalytic domain (amino acids 1–242) and an unstructured C-terminus (amino acids 243–314) implicated in membrane targeting^19^. TREX1 belongs to the DEDDh superfamily and contains an Asp-Glu-Asp-Asp motif and a conserved His residue that coordinate two metal ions and catalyze degradation of the 3′ end of DNA^20–22^. Elegant seminal studies with mouse TREX1 have provided a key model to explain enzymatic function and the role of mutations associated with autoimmune disease^11,12,22–29^. Human TREX1 (hTREX1) and mouse TREX1 (mTREX1) exhibit robust exonuclease activity in vitro, with each enzyme rapidly degrading double-stranded DNA with similar kinetics and capable of interacting with cGAS-DNA condensates to control immune activation^23,30^. However, hTREX1 and mTREX1 exhibit high sequence variation across evolution with hTREX1 containing 56 substitutions distributed throughout the N-terminal enzymatic domain suggesting altered regulation of enzymatic function. Additionally, >60 patient mutations in *TREX1* have been identified as associated with the autoimmune disease with many mutations occurring in hTREX1 positions not shared with mTREX1^17,31^. Although structures of mTREX1 continue to provide a critically important model for DNA degradation, these structures can only partially explain the role of human-specific variation in TREX1 regulation and TREX1-associated disease^22–27,30^.

Here, we determine a series of crystal structures of human TREX1 in the apo and DNA-bound conformations. We demonstrate that human-specific substitutions impact TREX1 protein stability and alter protein contacts with substrate DNA. Through an iterative chimera analysis approach, we systematically mapped molecular determinants of hTREX1 protein stability and DNA recognition and identify hyper-crystallizable variants for structural studies. Structures of human TREX1 and the human TREX1–DNA complex allow analysis of mutations associated with autoimmune disease and define 22 mutation sites predicted to directly impact human TREX1 function. We biochemically verify roles for specific hTREX1 substitutions in controlling TREX1–DNA complex formation and protein stability. Together our results reveal the structure of human TREX1 and establish a model to explain how human-specific substitutions regulate TREX1 activity and are impacted in TREX1-associated autoimmunity.

## Results

### Human-specific substitutions reduce TREX1 protein stability and prevent crystallization

**Fig. 1:**
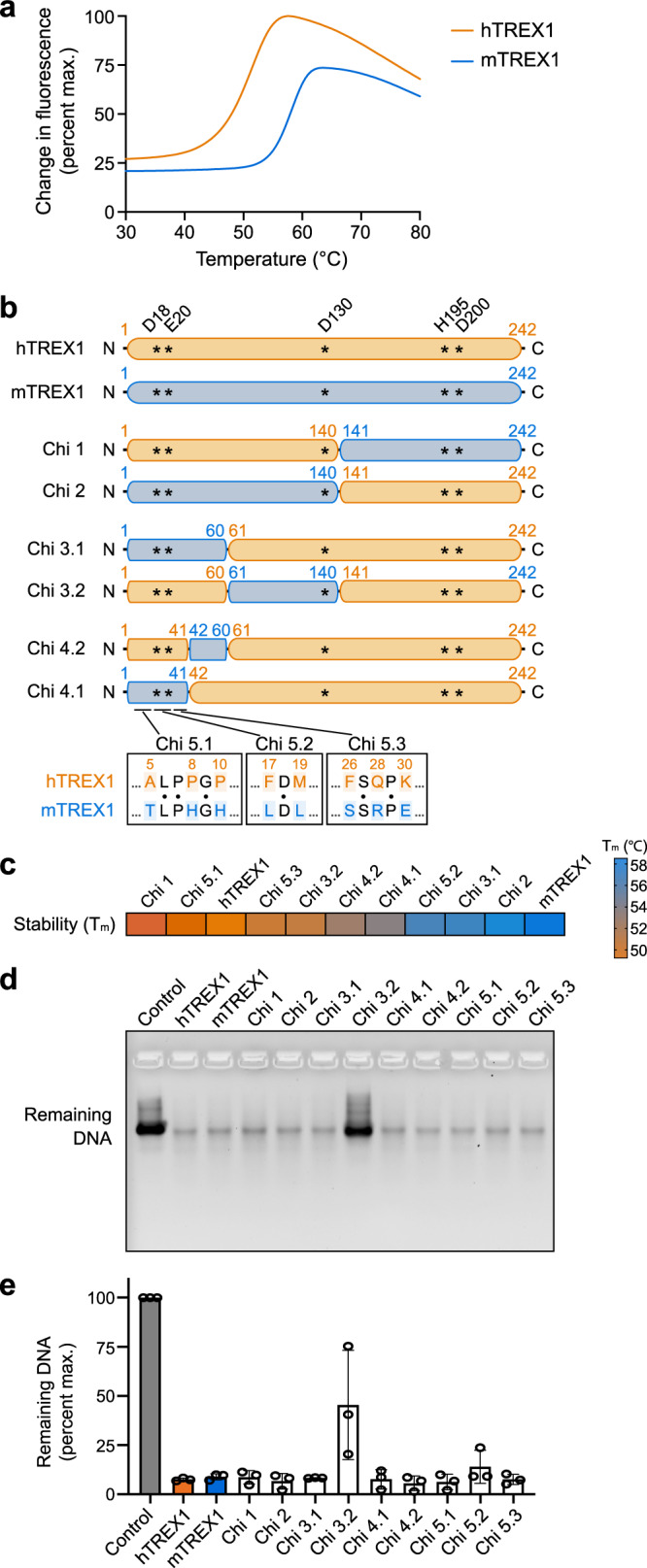
A biochemical screen to define the determinants of human TREX1 protein stability. **a** Thermal denaturation assay measuring thermal stabilization of N-terminal enzymatic domain of human and mouse TREX1. hTREX1 is less stable than mTREX1. Four independent experiments were performed and 1 single measurement was shown. Source data are provided as a Source Data file. **b** Schematic representation of chimeric and mutant TREX1 constructs. hTREX1 is colored in orange and mTREX1 is represented in blue. Asterisks indicate active sites of TREX1. Schematic is not to scale. **c** Heatmap of protein stability measured by thermal denaturation assay (see also Supplementary Fig. 2b). **d** In vitro analysis of DNA degradation by TREX1 chimeras and mutants. DNA degradation reactions were performed using 1 μM 100-bp double-stranded DNA (dsDNA) and 0.1 μM TREX1 proteins at 150 mM salt and the resulting reactions were resolved on 4% agarose gel. Data represent one of three independent experiments. Source data are provided as a Source Data file. **e** Quantification of DNA degradation by various TREX1 nucleases in **d**. Data are plotted as the mean ± SEM of 3 independent experiments. Source data are provided as a Source Data file. See also Supplementary Fig. 1 and 2.

To define how human-specific substitutions impact TREX1 function, we began by comparing the biochemical properties of purified human TREX1 (hTREX1) and mouse TREX1 (mTREX1) proteins in vitro. Thermofluor analysis demonstrated a significant ~7 °C difference in protein stability with hTREX1 exhibiting a melting temperature (T_m_) of 51.8 °C and mTREX1 exhibiting a T_m_ of 58.5 °C (Fig. 1a). Consistent with rapid evolution of mammalian innate immune proteins^32^, hTREX1 and mTREX1 are ~72% identical at the amino-acid level with 56 substitutions distributed throughout the enzymatic exonuclease domain (Supplementary Fig. 1). To determine if reduced protein stability is the result of specific substitutions in hTREX1 we designed a chimera-based genetic analysis approach to replace large sections of hTREX1 with mTREX1 sequence. Using the crystal structure of mTREX1 as a guide (PDB: 3MXJ), we divided the exonuclease domain after α-helix 5 and cloned two constructs exchanging the TREX1 N- or C-termini. hTREX1 Chi 1 containing the N-terminus of hTREX1 (amino acids 1–140) fused to the C-terminus of mTREX1 (amino acids 141–242) exhibited a T_m_ of 49.3 °C similar to that of the wildtype hTREX1 protein. In contrast, hTREX1 Chi 2 containing the N-terminus of mTREX1 fused to the C-terminus of hTREX1 exhibited a significant T_m_ shift to 58.0 °C demonstrating that N-terminal substitutions are responsible for reduced hTREX1 protein stability (Fig. 1b, c; Supplementary Fig. 2).

Protein thermal stabilization has an important role in controlling biological function^33,34^. To define the specific N-terminal substitutions controlling hTREX1 stability, we applied iterative rounds of TREX1 chimera design, protein purification, and thermofluor analysis (Fig. 1b, c). hTREX1 protein stability mapped to a region hTREX1 M1–R41 at the extreme N-terminus that contains 8 amino-acid substitutions compared to mTREX1 (Fig. 1b; Supplementary Fig. 2). Using hTREX1 Chi 4.1 as a background, we individually mutated each variant position and identified hTREX1 positions F17 and M19 as key determinants of protein stability. A hTREX1 Chi 5.2 construct containing mouse-like substitutions F17L and M19L exhibited a T_m_ of 57.0 °C, indicating that the difference in protein stability observed with hTREX1 is almost entirely attributable to these two positions in β-strand 1 (Fig. 1b, c; Supplementary Fig. 2). Most TREX1 chimeras exhibited robust DNA exonuclease activity with only subtle loss of activity observed for select chimeras not pursued further (Chi 3.2 and Chi 5.2) (Fig. 1d, e). Analysis with suboptimal reaction conditions revealed that in addition to impacting protein stability hTREX1 with F17L and M19L mutations (Chi 5.2) also exhibits slightly impaired TREX1 DNase activity in vitro (Supplementary Fig. 2e–g). Interestingly, F17 and M19 are specifically conserved in primates with L17 and L19 broadly present in lower mammals (Supplementary Fig. 2d). Together, these data reveal TREX1 protein stability as a biochemical property impacted by species-specific variation and define two substitutions that limit the protein stability of hTREX1.

### Structural analysis of human TREX1

**Fig. 2:**
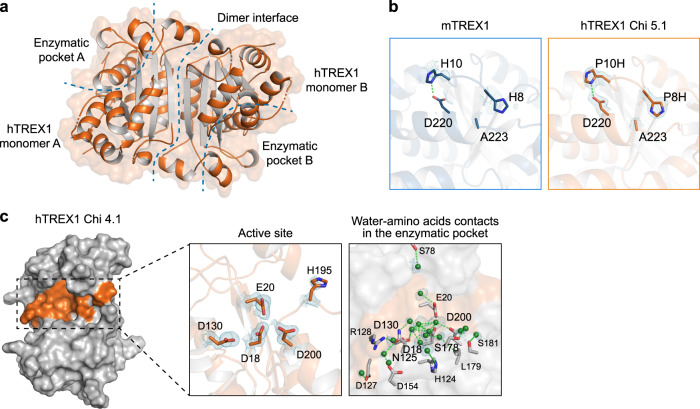
Structural analysis of human TREX1. **a** Overview of the human TREX1 dimer. Human TREX1 Chi 5.1 (A5T/P8H/P10H) organizes as two molecular dimer. The corresponding dimer interface and enzymatic pocket are highlighted. **b** Zoom-in cutaway showing location of site potentially needed for crystal formation. Similar to the observation in mTREX1 structural model, the P10H substitution hTREX1 makes direct contacts with C-terminal D220. This interaction may stabilize protein package and facilitate crystal formation. **c** Overview of a single hTREX1-unit in the hTREX1 dimer zoom-in cutaways of the TREX1 enzymatic pockets. The contacts of waters and amino acids in the core of enzymatic pocket is shown to the right as zoom-in cutaway. Green dots denote water molecules. 2Fo–Fc electron density map of water is highlighted (contoured at 1.0 σ). See also Supplementary Fig. 3.

All previous structural understanding of TREX1 function is derived from crystal structures of mTREX1^22–27,30^, limiting analysis of human-specific substitutions and the effects of patient mutations associated with autoimmune disease. Reasoning that reduced thermal stability and poor crystal packing may explain the lack of structural information for hTREX1, we screened each hTREX1 chimera for the ability for form protein crystals. We identified conditions for the growth of hTREX1 Chi 2, Chi 3.1, Chi 4.1, and Chi 5.1 crystals and determined three crystal structures (hTREX1 Chi 3.1, Chi 4.1, and Chi 5.1) (Supplementary Table 1). The hTREX1 structures include an exceptionally high-resolution 1.25 Å structure of hTREX1 Chi 4.1, and a 1.80 Å structure of hTREX1 Chi 5.1 that is 99.0% identical to the wildtype hTREX1 with only three mouse-like mutations (A5T, P8H, and P10H) in a single loop distal to the enzyme active site (Supplementary Fig. 3). hTREX1 Chi 5.1 P8H and P10H pack against C-terminal residues with P10H forming a salt-bridge with D220 that is also observed in mTREX1 structures suggesting that these mutations are directly responsible for facilitating packing and crystal formation (Fig. 2b).

The overall structure of hTREX1 adopts the homodimeric conformation previously defined for mTREX1 with enzymatic active sites on opposite outer faces of the dimer^22,23^ (Fig. 2a; Supplementary Fig. 3b). Solvation of an enzyme active site is a critical parameter for protein-small molecule interactions^35,36^. Notably, the high-resolution of the hTREX1 Chi 4.1 structure reveals the human catalytic active site in exceptional detail, including 18 well-defined waters in the core of the enzymatic pocket providing a template for structure-guided drug discovery (Fig. 2c).

### Mechanism of human TREX1 DNA recognition

**Fig. 3:**
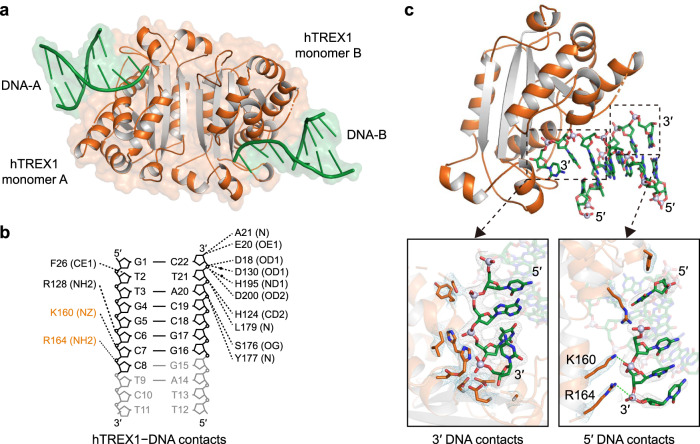
Mechanism of hTREX1-DNA recognition. **a** Overview of the human TREX1–DNA complex. Human TREX1 forms a 2:2 complex with DNA where each TREX1 monomer has one DNA binding surface. **b** Schematic map of protein–DNA contacts in the hTREX1–DNA complex. Human-specific contacts are highlighted in orange, and black dots denote interactions bridged by water molecules. See also Supplementary Fig. 4b. **c** Overview of a single 1:1 TREX1-unit in the hTREX1–DNA complex with zoom-in cutaways showing TREX1 to the 5′ and 3′ DNA contacts. The human-specific 5′ contacts by K160 and R164 are highlighted. See also Supplementary Fig. 4.

To define how human-specific substitutions impact TREX1 DNA recognition, we next sought to determine the structure of human TREX1 in complex with dsDNA. Using the hyper-crystallizable hTREX1 variants Chi 4.1, Chi 5.1, and a previously described mutant K66R^30^, along with a panel of 4 DNA substrates (including 24-bp dsDNA, 23-nt L-form dsDNA, 22-nt Y-form dsDNA, 19-nt L-from dsDNA) from published mTREX1–DNA structures^26,27^, we identified a diverse set of conditions that allowed the growth of hTREX1–DNA crystals. Remarkably, optimization around these initial conditions enabled crystallization of fully wildtype hTREX1 bound to Y-form dsDNA and we determined the structure of the wildtype hTREX1–DNA complex to 2.2 Å resolution (Fig. 3a and Supplementary Fig. 4a).

The hTREX1–DNA structure reveals a 2:2 complex of two molecules of TREX1 bound to two molecules of DNA with the 3′ end of each DNA threaded into the active site poised for degradation (Fig. 3a). hTREX1 makes extensive contact with both strands of DNA, including 10 residues that specifically recognize the 3′ end (D18, E20, A21, H124, D130, S176, Y177, L179, H195, D200) and 4 residues that contact the 5′ end DNA backbone (F26, R128, K160, and R164) (Fig. 3b, c). Comparative analysis with mTREX1–DNA structures^26,27^ revealed two human residues K160 and R164 that alter protein–DNA contacts at the DNA 5′ end (Fig. 3b and Supplementary Fig. 4b). hTREX1 K160 and R164 reside in α-helix 7 and make direct contact with the DNA phosphate backbone to stabilize the 5′ end (Fig. 3c). Notably, mutation of K160 is directly linked to autoimmune disease^37^, demonstrating that this substitution is likely critical for hTREX1 function. Interestingly, R164 only occurs in human and primate TREX1 sequences (Supplementary Fig. 4c), suggesting that hTREX1–DNA recognition is controlled by distinct contacts not shared with lower mammalian TREX1 homologs.

### Structural basis of human TREX1 autoimmune disease mutations

**Fig. 4:**
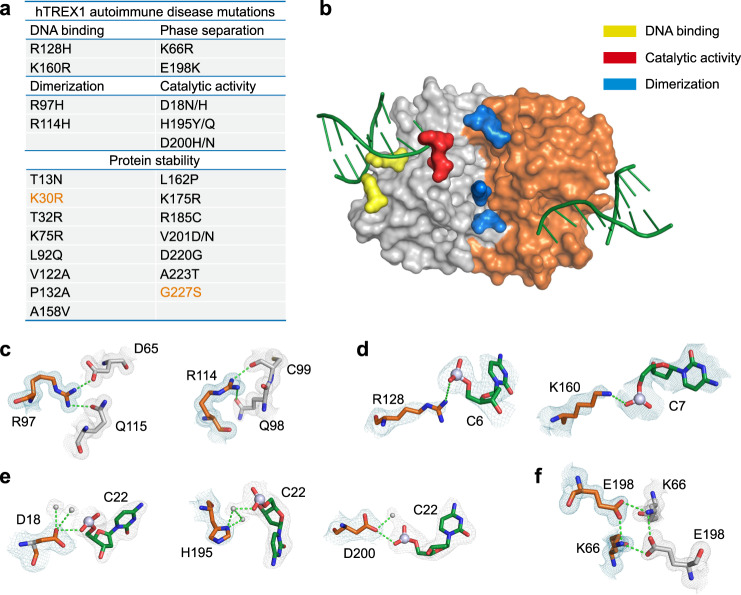
Structural basis of human TREX1 autoimmune disease mutations. **a** Autoimmune disease-associated mutations in TREX1, and the structural role of each residues predicted by the structures of hTREX1 *apo* and hTREX1–DNA complex. Residues that are not conserved between human and mouse TREX1 are highlighted in orange. **b** Highlights of the autoimmune disease-associated mutations in hTREX1 on the hTREX1–DNA complex. **c**–**f** Structural basis of TREX1 autoimmune disease mutations. The autoimmune disease related mutations, with the 2Fo−Fc map contoured at 1.0 are shown. Rely on the structure of hTREX1 and hTREX1–DNA complex, the mutations cause an interruption to dimerization (**c**), DNA binding (**d**), catalytic activity (**e**), or phase separation (**f**). The water molecules are depicted as gray spheres. See Supplementary Fig. 5 and 6.

High-resolution structures of hTREX1 in the *apo* and DNA-bound states allow systematic analysis of TREX1 mutations associated with autoimmune diseases, including Aicardi-Goutières syndrome, systemic lupus erythematosus, and familial chilblain lupus^7–12,38–40^. We first used the hTREX1 structures to analyze patient mutations and classify mutants into three categories that impact residues involved in protein dimerization, protein–DNA interactions, and active-site catalytic function (Fig. 4a, b). hTREX1 is an obligate dimer and patient autoimmune mutations R97H and R114H reside within the hTREX1 dimerization interface required for folding and enzymatic activity^20,22^ (Fig. 4c). R97 reaches across the dimer interface and forms hydrogen-bond contacts with D65 and Q115 on the opposite protomer. Likewise, hTREX1 R114 directly interacts with C99 and Q98 to stabilize the protein–protein interface. Two patient mutations R128H and K160R disrupt residues in the hTREX1–DNA structure that make contact the DNA phosphate backbone suggesting that these mutations cause disease by directly impairing DNA recognition (Fig. 4d). Finally, six hTREX1 mutations (D18N/H, H195Y/Q, D200H/N) occur in residues that form the enzymatic DEDDh active-site motif and are known to be essential for divalent metal ion coordination and catalysis^22^ (Fig. 4e).

Mapping disease-related residues onto the *apo* hTREX1 and hTREX1–DNA structures additionally reveals mutations that impact secondary properties of TREX1, including phase separation interactions and overall protein stability (Fig. 4a, b). We previously identified that the patient mutation E198K alters interactions with cGAS-DNA condensates^30^. In the hTREX1 structure, E198 hydrogen bonds with a second disease-related residue K66 demonstrating this interface is particularly sensitive to disruption (Fig. 4f). Several additional disease-related mutations, including T32R, R185C, and D220G influence electrostatics in the region surrounding E198, supporting that surface charge at this hTREX1 interface is critical for normal cellular function. We compared all remaining disease-related mutations side-by-side with the equivalent residues from previously determined mTREX1 structures. The hTREX1 electron density maps exhibit clear density for nearly all these positions and reveal a series of unique human-specific TREX1 contacts for residues K160, R114, H195, T13, K30, T32, L92, P132, L162, and K175 (Fig. 4c–f, Supplementary Figs. 5 and 6). Many residues including T13, K30, and K75 are predicted to be required for overall protein stability, while some positions, including P132, A158, and A223 are of unknown functional importance.

**Fig. 5:**
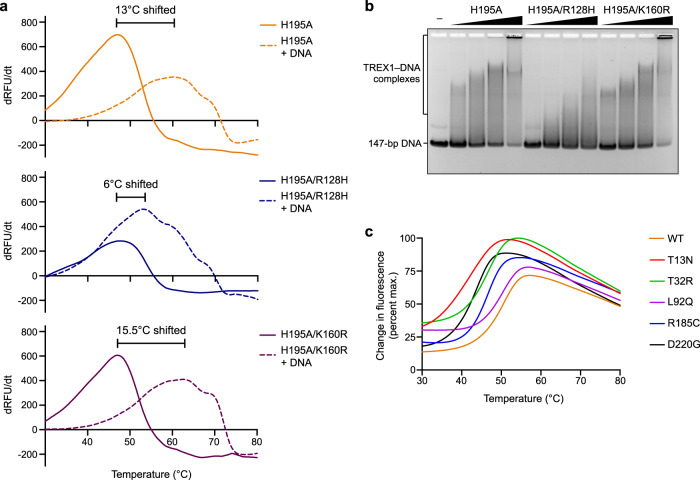
Analysis of hTREX1 substitutions on DNA-binding and protein stability. **a** Thermofluor analysis melting curves measuring hTREX1-DNA stable complex formation. Disease-associated mutations R128H and K160R exhibit abnormal DNA-binding compared to WT hTREX1. Data are representative of at least 3 independent experiments. Source data are provided as a Source Data file. See also Supplementary Fig. 7c. **b** In vitro electrophoretic mobility shift assay measurement of TREX1–DNA complex formation. Data are representative of 3 independent experiments. Source data are provided as a Source Data file. **c** Thermal denaturation assay to quantify the protein stability of TREX1 disease-associated mutants, as quantified in Supplementary Fig. 7d. Data are representative of 4 independent experiments. Source data are provided as a Source Data file. See Supplementary Fig. 7.

To begin to define the role of individual TREX1 substitutions, we purified and tested seven hTREX1 variants with mutations structurally predicted to impact DNA binding (R128H, K160R) or protein stability (T13N, L92Q, T32R, R185C, D220G) (Fig. 4a). Compared to wildtype hTREX1, hTREX1 R128H exhibited impaired protein–DNA complex formation (Fig. 5a, b; Supplementary Fig. 7). In contrast, hTREX1 K160R exhibited a higher thermal-stability in the presence of DNA suggesting that similar to previous experiments with mTREX1 that mutations that enhance DNA binding may be disease-associated due to impacting interactions between TREX1 and cGAS-DNA condensates^30^ (Fig. 5a, b; Supplementary Fig. 7). hTREX1 T13N, T32R, R185C, and D220G substitutions predicted to impact protein stability each exhibited a T_m_ reduction of 4–8 °C in vitro, while no stability defect was observed for L92Q (Fig. 5c; Supplementary Fig. 7). Together, these results highlight how structures of hTREX1 advance understanding of TREX1 biochemical function and human TREX1-associated autoimmune disease.

## Discussion

High-resolution structures of human TREX1 and the hTREX1–DNA complex provide a new foundation to understand cytosolic DNA degradation and regulation of innate immune sensing. Previous studies on the structures of mouse TREX1 have been instrumental in establishing the conserved mechanism of TREX1-DNA recognition, dimerization and DNA degradation^22–27^. Building on the key advances of these mTREX1 structures^22–27,30^, our data now allow direct analysis of the importance of human sequence variation on TREX1 enzymatic function. Notably, the hTREX1 structure and our biochemical results suggest human-specific substitutions play a role in modulating TREX1 stability and DNA substrate recognition. We define two substitutions F17 and M19 that occur only in human and primate TREX1 sequences (Supplementary Fig. 2) and cause reduced thermal stability and exonuclease activity compared to mTREX1. F17 and M19 residues reside in TREX1 β-strand 1 close to amino acids critical for DNA recognition and cleavage suggesting additional possible roles for this region of the protein in regulating human TREX1 function. Additionally, the structure of hTREX1–DNA complex reveals 14 residues that specifically coordinate substrate DNA including two human residues K160 and R164 that make direct contact with the DNA 5′ end (Fig. 3 and Supplementary Fig. 4). Mutations to K160 and this hTREX1 surface have been implicated in dsDNA unwinding explaining how DNA 5′ end contacts modulate DNA exonuclease activity^26,27,37^. The occurrence of human- and primate-specific TREX1 substitutions in residues directly linked to protein stability and DNA recognition suggests that positive-selection may control regulation of TREX1 function similar to human-specific substitutions that alter the ability of cGAS to recognize cytosolic DNA^41,42^. Details of the biological roles for human-specific substitutions remain an important direction for future investigation.

The structure of human TREX1 additionally enables direct analysis of the relationship between TREX1 mutations and autoimmune disease. Our mapping of reported TREX1 disease-associated mutations demonstrates 22 patient mutation sites that are predicted to impact human TREX1 protein stability, dimerization, DNA substrate recognition, and catalytic function (Figs. 4, 5 and Supplementary Figs. 5–7). Autoimmune disease-associated TREX1 mutations in surface-exposed residues (e.g., E198K, K66R, P132A, A223T) may also alter binding to protein partners, post-translational modifications, and interaction with cGAS-DNA condensates^30,37,43^. In addition to association with autoimmune disease, recently reported links between TREX1 dysregulation and cancer suggest inhibition of TREX1 exonuclease activity is a promising target for cancer immunotherapy^14–18^. High-resolution structures of hTREX1 and hyper-crystallizable hTREX1 variants now provide key insight and tools to facilitate the development of TREX1 therapeutics.

## Methods

### *Escherichia coli* strains, protein expression and purification

Recombinant TREX1 proteins (TREX1 C-terminal truncation, amino acid numbering 1–242) were expressed in *Escherichia coli* (*E. coli*) BL21-RIL DE3 (Agilent) bacteria harboring a pRARE2 tRNA plasmid. Transformations and starter cultures were grown in 30 mL of MDG media (25 mM Na_2_HPO_4_, 25 mM KH_2_PO_4_, 50 mM NH_4_Cl, 5 mM Na_2_SO_4_, 2 mM MgSO_4_, 0.5% glucose, 0.25% aspartic acid, 100 mg mL^−1^ ampicillin, 34 mg mL^−1^ chloramphenicol, and trace metals) overnight at 37 °C, and were used to seed 1–2 L M9ZB media cultures (47.8 mM Na_2_HPO_4_, 22 mM KH_2_PO_4_, 18.7 mM NH_4_Cl, 85.6 mM NaCl, 2 mM MgSO_4_, 0.5% glycerol, 1% Cas-amino acids, 100 mg mL^−1^ ampicillin, 34 mg mL^−1^ chloramphenicol, and trace metals). M9ZB media cultures were then grown at 37 °C until OD_600_ of 1.5–2.5. After cooling on ice for ~20 min, recombinant TREX1 expression was induced by supplementation with 0.5 mM IPTG into bacterial cultures. Next, cultures were incubated at 16 C° with shaking at 230 RPM for ~16 h before harvest. Bacteria cultures were subsequently pelleted, flash-frozen in liquid nitrogen, and stored at −80 °C until purification.

TREX1 proteins were purified as previously described^30,42^. Briefly, bacterial pellets were re-suspended in lysis buffer (20 mM HEPES-KOH pH 7.5, 400 mM NaCl, 10% glycerol, 30 mM imidazole, 1 mM DTT) and then lysed by sonication. After centrifugation, the supernatant was collected for purification. The initial purification was performed using Ni-NTA (Qiagen) resin and gravity chromatography. The elution fraction of TREX1 protein from Ni-NTA was supplemented with ~250 μg of human SENP2 protease to remove the His–SUMO2 tag and dialyzed in dialysis buffer (20 mM HEPES-KOH pH 7.5, 150 mM NaCl, 1 mM DTT) at 4 °C for ~12 h. Next, untagged TREX1 was purified using Heparin HP ion-exchange (Cytiva) and eluted with a gradient of 150–1000 mM NaCl. TREX1 protein was further purified with size-exclusion chromatography using a 16/600 Superdex S75 column (Cytiva). TREX1 was collected in storage buffer (20 mM HEPES-KOH pH 7.5, 250 mM KCl, 1 mM TCEP). Finally, the purified TREX1 was concentrated to ~10 mg mL^−1^, flash-frozen in liquid nitrogen, and stored as aliquots at −80 °C for biochemical experiments and crystallization.

### Thermal denaturation assay

10 μM TREX1 or TREX1 chimeras was supplemented with 3× SYPRO Orange Dye (Life Technologies) in a 20 μL reaction buffer containing 20 mM Tris-HCl pH 7.5, 75 mM KCl, and 1 mM TCEP. Reactions were incubated with an increasing temperature from 20 to 95 °C over ~2 h using a qPCR CFX96 thermocycler (Bio-Rad). Fluorescence in the HEX channel was measured every 0.5 °C and melting temperature (T_m_) was defined as the temperature at which the half of the maximum fluorescence change occurs. For TREX1-DNA binding analysis, 10 μM TREX1 or TREX1 mutants was incubated with a 30-bp dsDNA (10 or 20 μM; sequence: 5′-GCTCGAGTCATGACGCGTCATGACTCGAGC-3′) at 4 °C for 30 min, and the reactions were subjected for thermal denaturation analysis as previously describe^44^.

### In vitro electrophoretic mobility shift assay (EMSA)

In vitro measurement of TREX1–DNA complex formation by EMSA was performed as previously described for cGAS–DNA complex with minor modifications^42^. Briefly, 0.5 μM 147-bp dsDNA was incubated with hTREX1 H195A, hTREX1 H195A/R128H, or hTREX1 H195A/K160R at a gradient of protein concentrations (1, 2, 4, 8 μM) in a 20 μL EMSA reaction buffer (20 mM HEPES-NaOH pH 7.8, 75 mM KCl, 10 mM EDTA, 1 mM DTT) at 4 °C for 30 min. The resulting reactions were then separated on a 2% agarose gel in 0.5 × TB buffer as a running buffer. After electrophoresis, the agarose gel was further stained in 0.5 × TB buffer containing 10 μg mL^−1^ ethidium bromide. The TREX1-DNA complex was visualized with a AI800 Imaging Systems (Cytiva).

### In vitro TREX1 DNA degradation assays

TREX1 DNA degradation assays were conducted as previously described with minor modifications^30^. Briefly, 0.1 μM TREX1 or TREX1 chimera protein was incubated with a 100 bp double-stranded DNA (see below) in the 20 μL reaction system containing 20 mM Tris-HCl pH 7.5, 15 mM NaCl, 135 mM KCl, 5 mM MgCl_2_, and 1 mg mL^−1^ BSA at 25 °C for 30 min. DNA degradation was terminated by adding 10 mM EDTA and incubating at 70 °C for 15 min. Reactions were then separated on a 4% agarose gel using chilled 0.5× TB buffer as a running buffer. After electrophoresis, the gel was stained with 0.5× TB buffer containing 10 μg mL^−1^ ethidium bromide at 25 °C for 45 min, and then de-stained with milli-Q water at 25 °C for 10 min. DNA was visualized with a ChemiDoc MP imaging System (Bio-Rad) and quantified via FIJI^45^.

100 bp dsDNA sense: 5′-ACATCTAGTACATGTCTAGTCAGTATCTAGTGATTATCTAGACATACATCTAGTACATGTCTAGTCAGTATCTAGTGATTATCTAGACATGGACTCATCC -3′

100 bp dsDNA anti-sense: 5′- GGATGAGTCCATGTCTAGATAATCACTAGATACTGACTAGACATGTACTAGATGTATGTCTAGATAATCACTAGATACTGACTAGACATGTACTAGATGT -3′

### Crystallization and Structure Determination

Determined hTREX1 structures include hTREX1 Chi 3.1 *apo*, hTREX1 Chi 4.1 *apo*, hTREX1 Chi 5.1 *apo*, and the hTREX1–DNA complex. For hTREX1 Chi 3.1 *apo* crystallization, purified hTREX1 Chi 3.1 was diluted to 16 mg mL^−1^ in a final buffer containing 20 mM HEPES-KOH pH 7.5, 128 mM KCl, and 1 mM TCEP. hTREX1 Chi 3.1 crystals were obtained with hanging-drop vapor diffusion in drops mixed 1:1 over a reservoir of 0.2 M NaCl, 0.1 M CHES pH 9.5, 1.26 M (NH_4_)_2_SO_4_ after 1 day of growth at 18 °C, and were cryo-protected with NVH oil (Cargille) prior to freezing in liquid nitrogen. For hTREX1 Chi 4.1 *apo* crystallization, purified hTREX1 Chi 4.1 was diluted to 14 mg mL^−1^ in a final buffer consisting of 20 mM HEPES-KOH pH 7.5, 63 mM KCl, and 1 mM TCEP. hTREX1 Chi 4.1 crystals were grown in hanging-drop format in drops mixed 1:1 over a reservoir of 0.1 M HEPES-KOH pH 7.6, 10% PEG-8000 after 3 days of growth at 18 °C, and were then frozen in liquid nitrogen without cryo-protectant. For hTREX1 Chi 5.1 *apo* crystallization, purified hTREX1 Chi 5.1 was diluted to 16 mg mL^−1^ in a final buffer containing 20 mM HEPES-KOH pH 7.5, 63 mM KCl, and 1 mM TCEP. Optimized hTREX1 Chi 5.1 crystals were grown in hanging-drop format using 15-well EasyXtal trays (Qiagen) in 2 μL drops containing a 1:1 mixture of protein solution and reservoir solution (0.1 M Sodium chloride, 0.1 M Bis-Tris pH 5.5, 20% PEG-3350). After the growth for 3 days at 18 °C, crystals were cryo-protected using reservoir solution supplemented with 10% glycerol and flash-frozen in LiN_2_. For crystallization of hTREX1 with DNA, 22-nt Y-form dsDNA oligonucleotide sequence was used as previously describe for mTREX1^27^. Purified WT hTREX1 (final 11 mg mL^−1^) was mixed with 22-nt Y-form dsDNA in a molar ratio of 1:1.3 protein:DNA in a buffer containing 20 mM HEPES-KOH pH 7.5, 78 mM KCl, and 1 mM TCEP. Crystals of hTREX1–DNA complex were obtained with hanging drop vapor diffusion in 2 μL drops mixed 1.2:0.8 over a reservoir of 0.05 M Li_2_SO_4_, 0.1 M Bis-Tris pH 6.5, 25% PEG-3350 after 3 days growth at 18 °C. Crystals were then cryo-protected using reservoir solution with additional 10% EtGl and flash-frozen in LiN_2_.

X-ray diffraction data were collected at the Northeastern Collaborative Access Team (beamline 24-ID-E, P30 GM124165) and used an Eiger detector (S10OD021527) and the Argonne National Laboratory Advanced Photon Source (DE-AC02-06CH11357). X-ray data were then processed with XDS and AIMLESS^46^ using the SSRL *autoxds* script (A. Gonzalez, Stanford SSRL). Crystals for all *apo* hTREX1 were indexed according to the tetragonal spacegroup *P* 4_1_ 2_1_ 2 and contain one copy of a hTREX1 dimer in the asymmetric unit. Crystals for hTREX1–DNA complex were indexed according to the monoclinic spacegroup *P* 1 2_1_ 1 and contain 3 copies of hTREX1–DNA dimer in the asymmetric unit. Phases were determined with molecular replacement using Phaser-MR in PHENIX^47^ and the *apo* mTREX1 structure (PDB: 3MXJ) as a search model for hTREX1 Chi 4.1. The structure of *apo* hTREX1 Chi 4.1 was then used as a search model for Chi 3.1, Chi 5.1 and hTREX1–DNA complex. Model building and structural determination were completed with Coot^48^ and PHENIX^49–51^. Details of data collection and refinement statistics are listed in Supplementary Table 1.

### Statistics and reproducibility

Details of quantification and statistical analysis are listed in the Figure legends. Experiments were performed with at least 3 independent replicants. Statistical analyses were conducted using Graphpad Prism Version 9.1. Data are plotted with error bars representing standard error of the mean (SEM).

### Reporting summary

Further information on research design is available in the Nature Research Reporting Summary linked to this article.

## Supplementary information

Supplementary Information

Reporting Summary

## Data Availability

Coordinates of the TREX1 structures have been deposited in the RCSB Protein Data Bank under accession numbers 7TQN [10.2210/pdb7tqn/pdb], 7TQO [10.2210/pdb7tqo/pdb], 7TQP [10.2210/pdb7tqp/pdb], and 7TQQ [10.2210/pdb7tqq/pdb]. The authors declare that all other data supporting the findings of this study are available within the paper and Supplementary information or from the corresponding authors upon request. Source data (underlying Figs. 1a,d,e, 5a,b,c and Supplementary Figs. 2a,b,e,f,g,7a,b,c,d) are provided with this paper. Source data are provided with this paper.

## Source data

Source Data
