# Supplementary material for: Structural basis of human TREX1 DNA degradation and autoimmune disease
Source: Nat Commun. 2022 Jul 25;13:4277. doi: 10.1038/s41467-022-32055-z (PMC9314330; doi:10.1038/s41467-022-32055-z)
Supplement: Supplementary file 1 — Supplementary Information [file 41467_2022_32055_MOESM1_ESM.pdf]

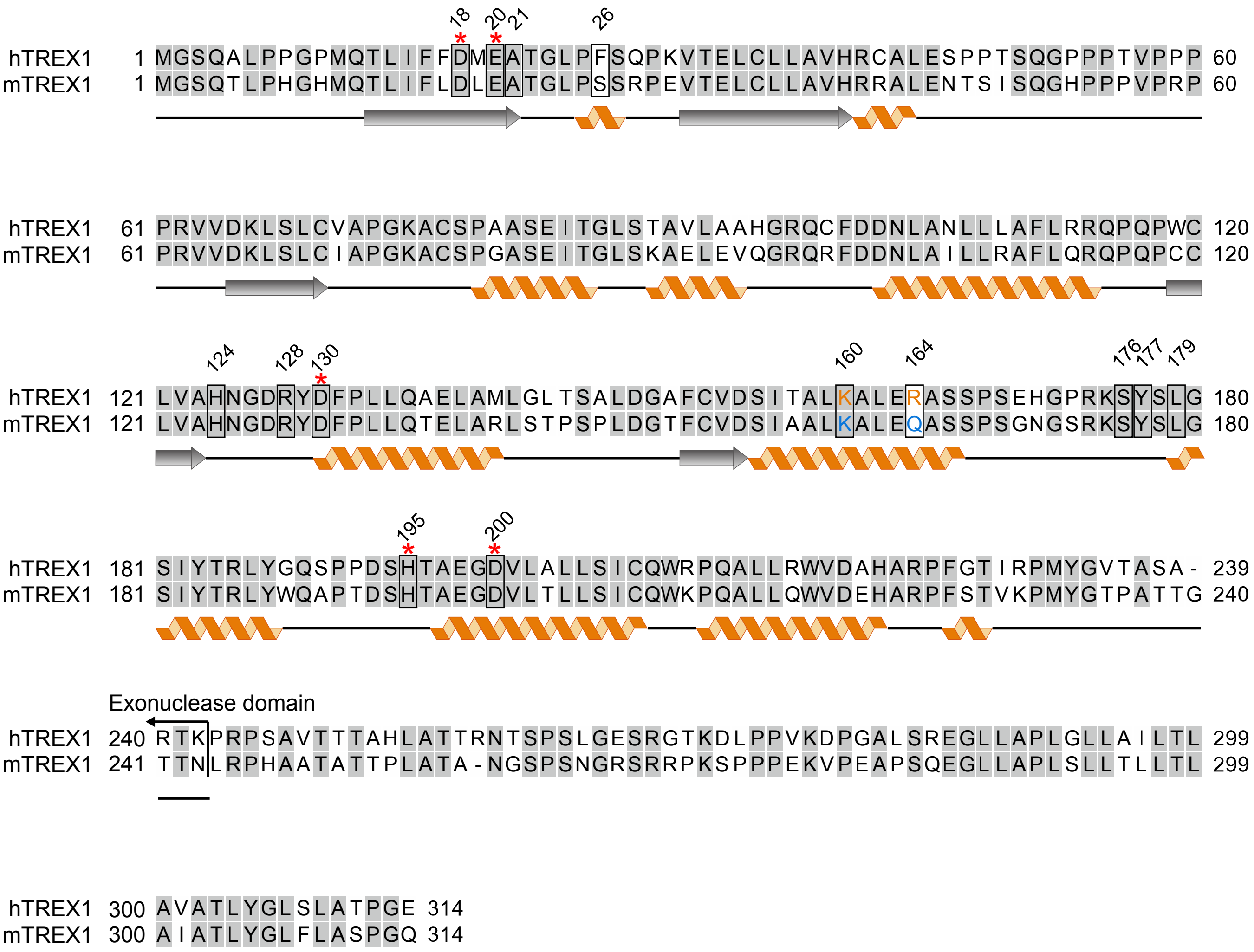

Supplementary Figure 1. Sequence alignment of hTREX1 and mTREX1, related to Fig. 1

Alignment of human and mouse TREX1 amino acid sequences with alpha-helix (orange) and beta-strand (gray) secondary structure depicted below. Conserved residues are shaded in gray, the active site residues are marked with red asterisks. The amino acids contributed to hTREX1–DNA contacts are boxed in black. Human-specific DNA contacts are labeled in orange and corresponding mouse residues are labeled in blue.

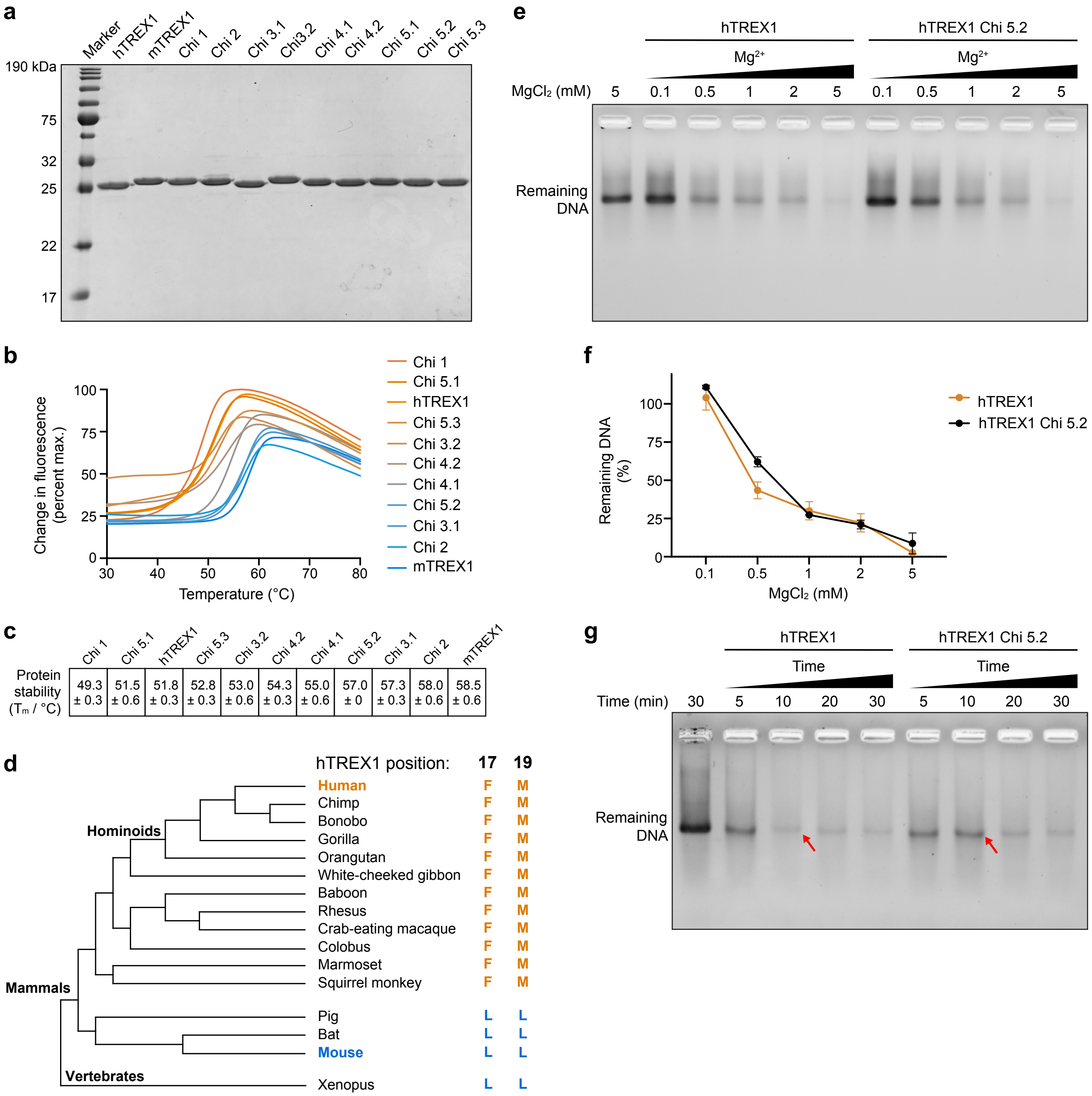

Supplementary Figure 2. Protein purification and thermal denaturation assay, related to Fig. 1

(a) Purified hTREX1 and mTREX1 nucleases used in this study. Purified recombinant TREX1 (~1 μg each) was resolved on a denaturing SDS-PAGE gel and visualized with Coomassie G-250 stain. All TREX1 nucleases were purified with Ni-NTA, heparin ion-exchange, and Superdex S75 size-exclusion chromatog-raphy. Source data available in Source Data file.

(b) Thermal denaturation assay measuring thermal stabilization of various TREX1 proteins. Four independent experiments were conducted and data represent 1 single experiment. Source data available in Source Data file.

(c) Tm values of TREX1 proteins. Data are representative of 4 independent experiments.

(d) Cladogram depicting evolution of hTREX1 F17 and M19 in primates and relevant vertebrates. Human-specific substitutions are denoted un orange, and the mTREX1-like sequence is denoted in blue.

(e) *In vitro* analysis of DNA degradation by hTREX1 and Chi 5.2 with a gradient of Mg^{2+} concentrations. Data are representative of 4 independent experiments. Source data available in Source Data file.
(f) Quantification of DNA degradation by TREX1 enzymes in (e). Data are plotted as the mean ± SEM of 4 independent experiments. Source data available in Source Data file.
(g) *In vitro* analysis of DNA degradation by hTREX1 and Chi 5.2 with a gradient of reaction time. The red arrows indicate Chi 5.2 degrades DNA more slowly compared to WT enzyme at the reaction time of 10 min. Data are representative of at least 3 independent experiments. Source data available in Source Data file.

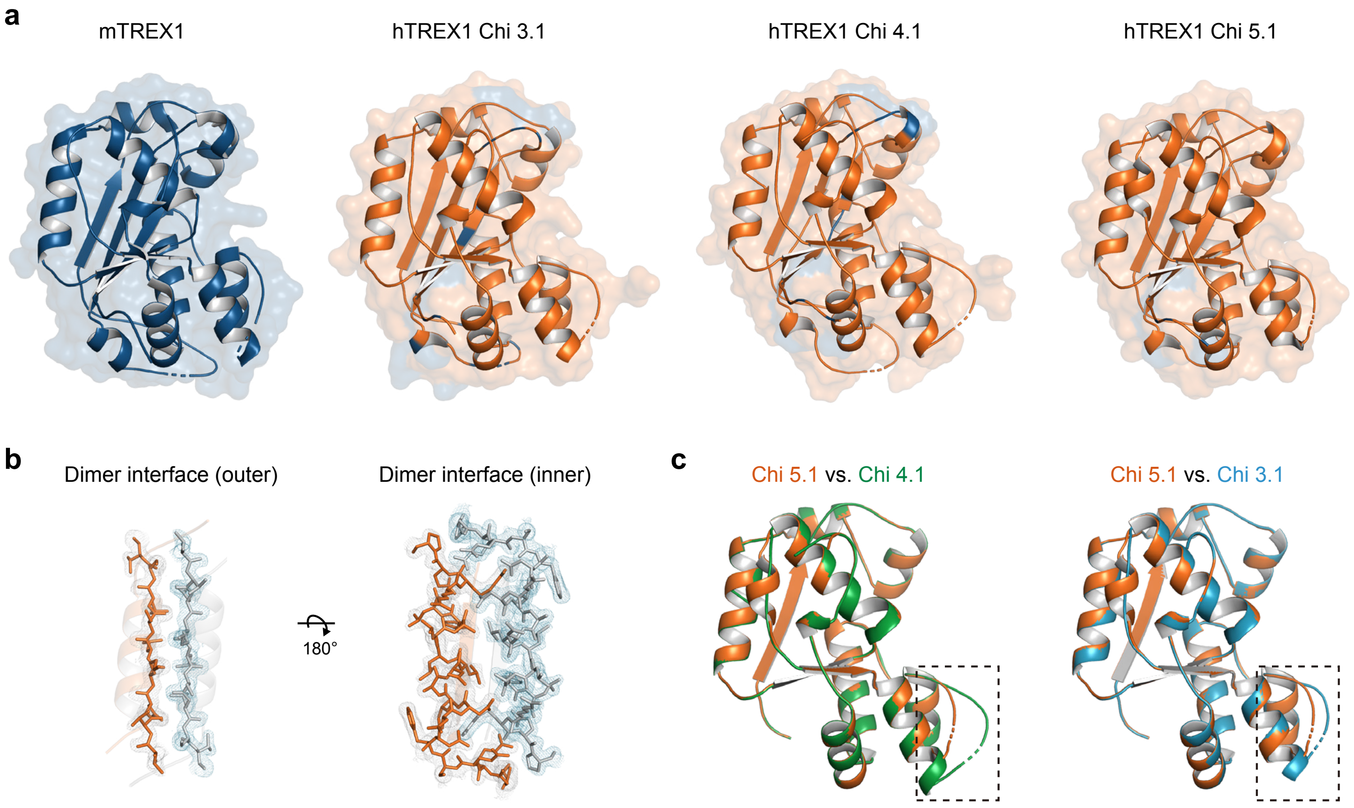

Supplementary Figure 3. Structure models of hTREX1 chimeras (Chi 3.1, 4.1 and 5.1), related to Fig. 2

(a) Overview of structural models of mTREX1, hTREX1 Chi 3.1, Chi 4.1 and Chi 5.1 (from left to right). Monomer TREX1 models were shown (PDB: 3MXJ). The amino acids from mouse and human TREX1 are labeled in blue and orange, respectively. Structure superposition reveals corresponding RMSD values for hTREX1 Chi 5.1 and Chi 4.1 (0.155 Å), Chi 5.1 and Chi 3.1 (0.190 Å), and Chi 4.1 and Chi 3.1 (0.166 Å).
(b) Zoom-in cutaways of the TREX1 dimerization surface (hTREX1 Chi 5.1 model at 1.80 Å).
(c) Superposition of hTREX1 Chi 5.1 and Chi 4.1, and Chi 5.1 and Chi 3.1. *Left*, overview of Chi 5.1 (residues 61–242) superposed with Chi 4.1 (residues 61–242) with an RMSD of 0.149 Å, and *Right*, with Chi 3.1 (residues 61–242) with Chi 5.1 (orange) with an RMSD of 0.187 Å. Chi 4.1 (green), and Chi 3.1 (blue) are shown as cartoon. Structural difference is highlighted with a dashed box.

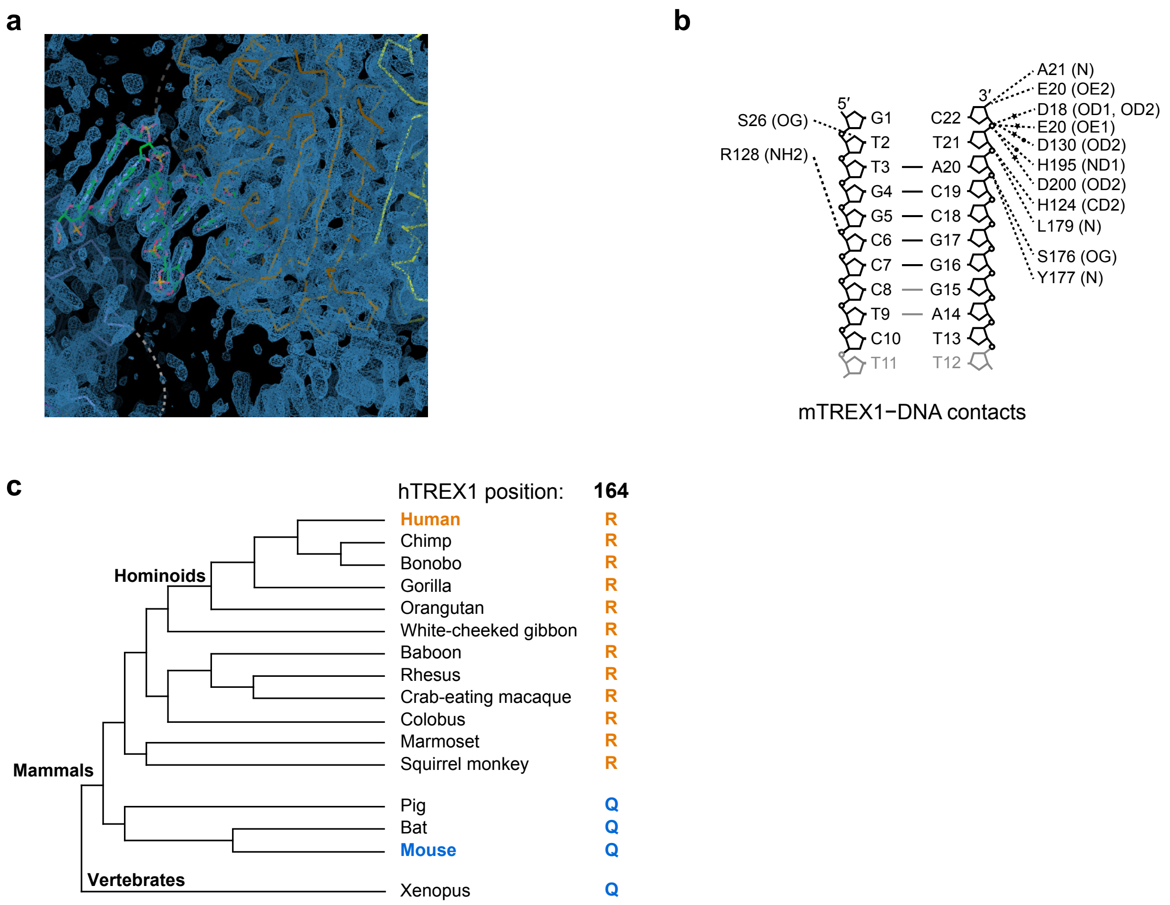

Supplementary Figure 4. Mechanism of hTREX1-DNA recognition, related to Fig. 3

(a) 2Fo−Fc electron density map of the 2.2 Å hTREX1-DNA complex (contoured at 1.0 σ). hTREX1 is shown as a ribbon (orange/yellow) and the DNA is shown as sticks (green).

(b) Schematic map of protein-DNA contacts in the mTREX1–DNA complexes (PDB 5YWS). Black dots denote interactions bridged by water molecules, and star indicates metal magnesium.

(c) Cladogram depicting evolution of hTREX1 R164 in primates and relevant vertebrates. Human-specific substitutions are denoted un orange, and the mTREX1-like sequence is denoted in blue.

a

Human TREX1	Side-chain contacts	Backbone contacts	Mouse TREX1	Side-chain contacts	Backbone contacts
T13	P116	P116	T13	P116	
K30	E137		E30	R99	
T32	T85	E20, A21	T32	T85	A21
K75		S27, R28	K75		S27, R28
L92		V71	L92		
P132		G126, Y129, R230	P132		G126
L162		A158	L162		
K175	E163		K175		
R185	E163	S176	R185	E163	S176
D220	H10, R41	M11	D220	H10, R41	M11

b

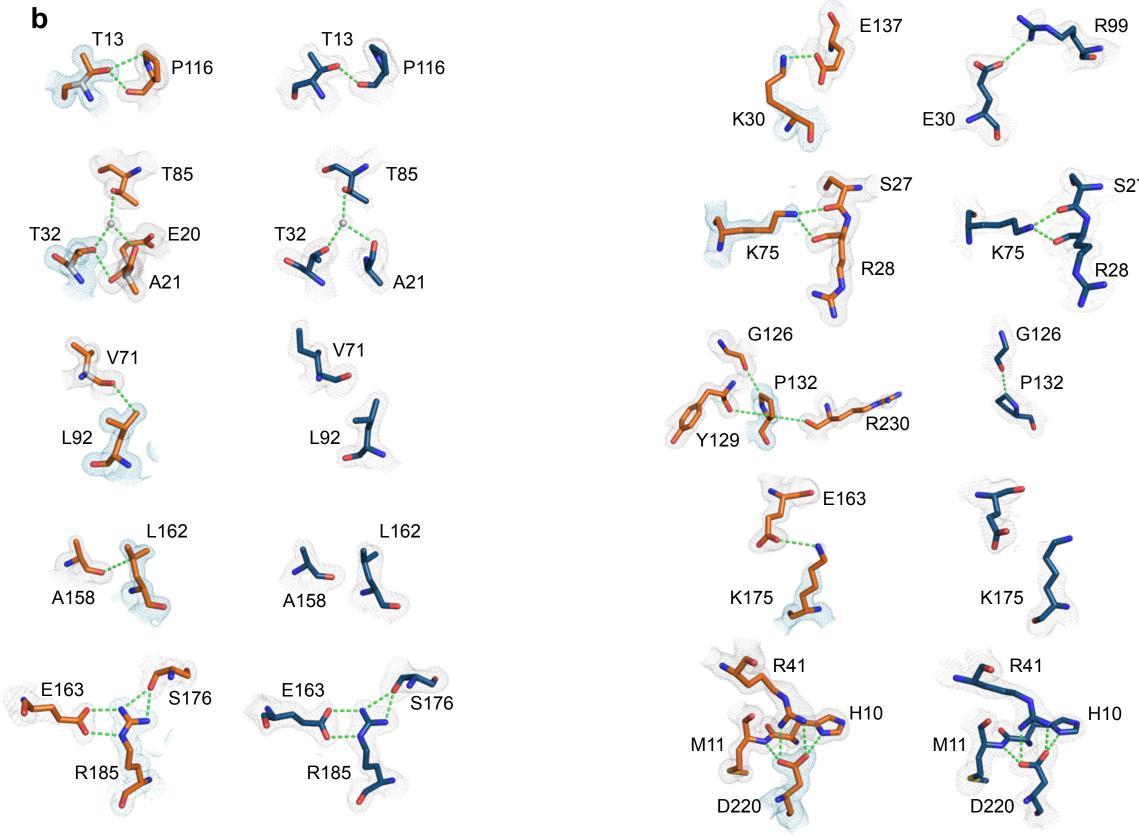

Supplementary Figure 5. Structural analysis of hTREX1 disease-related mutations predicted to impact protein stability, related to Fig. 4

(a) Side-chain and backbone contacts of residues required for overall protein stability as predicted by the hTREX1 apo structure (Chi 4.1) and mTREX1 apo structure (PDB: 3MXJ).

(b) 2Fo−Fc electron density maps (contoured at 1.0σ) of each residue in (a). hTREX1 is highlighted as sticks in orange and mTREX1 is denoted in blue. Water molecules are depicted as gray spheres.

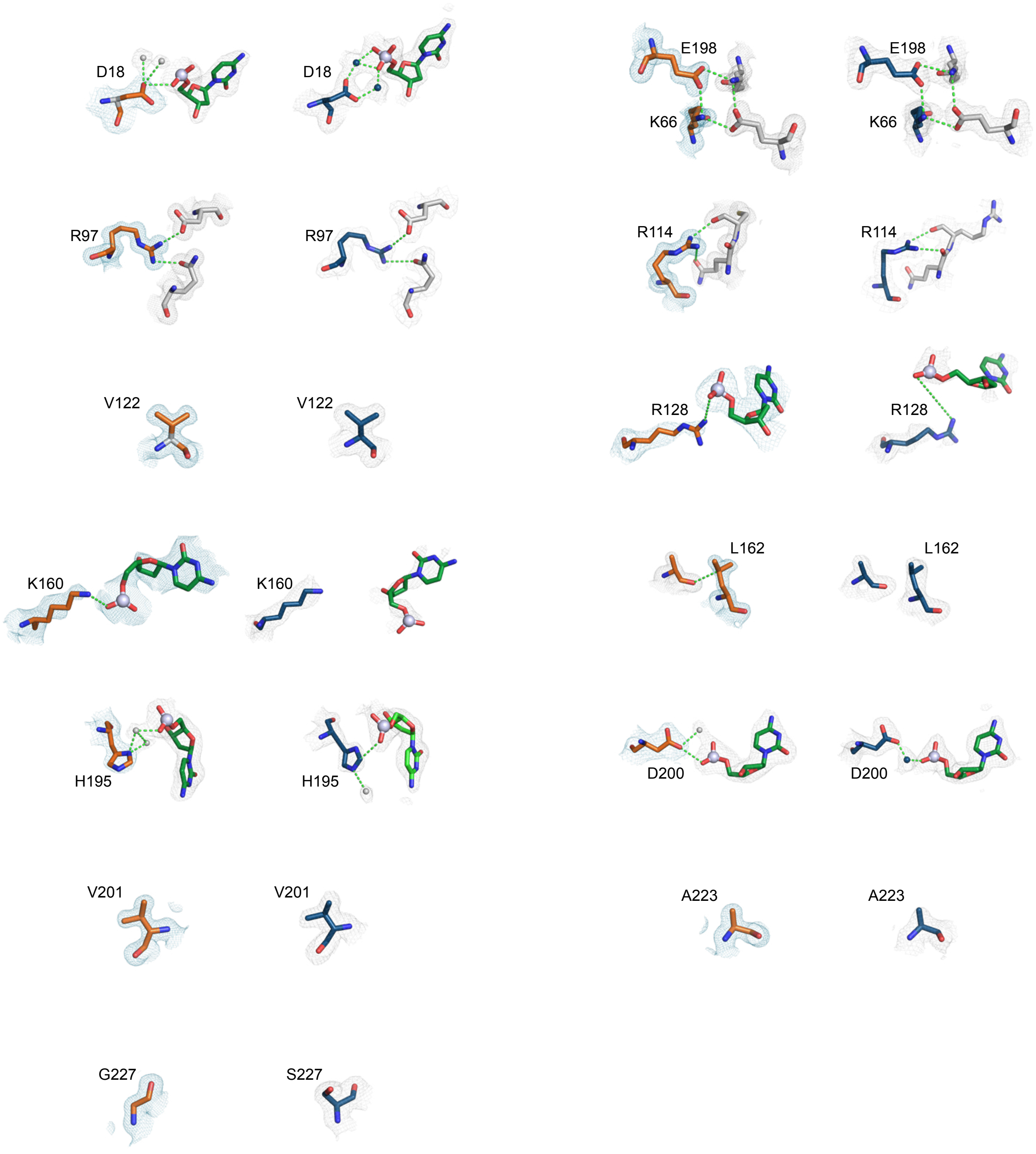

Supplementary Figure 6. Side-by-side comparisons of hTREX1 and mTREX1 structures, related to Fig. 4

hTREX1 is highlighted as sticks in orange and mTREX1 is denoted in blue. The 2Fo−Fc electron density map is contoured at 1.0σ. Water molecules are depicted as gray spheres and Mg^{2+} are labeled as blue spheres.

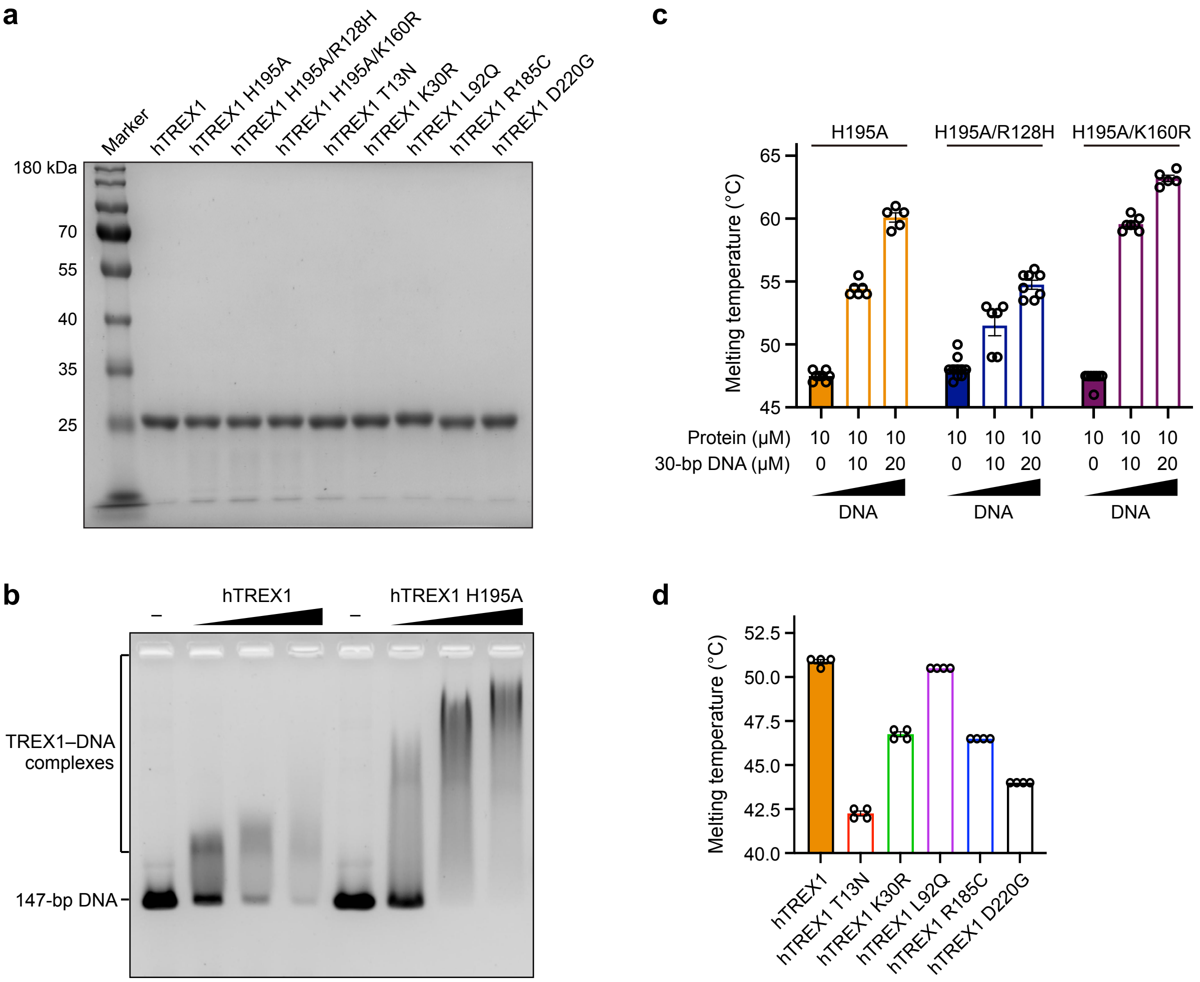

Supplementary Figure 7. Experimental validations of the structural basis of disease-related mutations involved in protein stability, related to Fig. 5

(a) Purified hTREX1 and disease-associated mutant proteins used in this study. Source data available in S ource Data file.

(b) *In vitro* electrophoretic mobility shift assay demonstrating that the catalytically dead mutant (H195A) but not WT hTREX1 is suitable for TREX1-DNA binding analysis. Data are representative of 2 independent experiments. Source data available in Source Data file.

(c) Thermal denaturation assay to quantify DNA binding affinity of TREX1 mutants. Data are plotted as the mean ± SEM of at least 5 independent experiments. Source data available in Source Data file.

(d) Thermal denaturation assay to quantify the stability of TREX1 mutants. Data are plotted as the mean ± SEM of 4 independent experiments. Source data available in Source Data file.

Supplementary Table 1. Crystallographic Statistics

	hTREX1 Chi 3.1	hTREX1 Chi 4.1	hTREX1 Chi 5.1	hTREX1–DNA
**Data Collection**				
Resolution (Å)[a]	42.47–1.95 (2.00–1.95)	42.26–1.25 (1.27–1.25)	41.86–1.80 (1.84–1.80)	45.68–2.20 (2.28–2.20)
Wavelength (Å)	0.9792	0.9792	0.9792	0.9792
Space group	$P\ 4_1\ 2_1\ 2$	$P\ 4_1\ 2_1\ 2$	$P\ 4_1\ 2_1\ 2$	$P\ 1\ 2_1\ 1$
Unit cell: a, b, c (Å)	57.58, 57.58, 125.76	57.59, 57.59, 124.40	57.08, 57.08, 123.12	43.68, 91.36, 157.39
Unit cell: α, β, γ (°)	90.0, 90.0, 90.0	90.0, 90.0, 90.0	90.0, 90.0, 90.0	90.0, 90.7, 90.0
Molecules per ASU	1	1	1	4
No. reflections: total	349639	2689137	255740	343037
No. reflections: unique	16216 (1098)	58943 (2773)	19529 (1133)	62516 (4576)
Completeness (%)[a]	99.9 (98.8)	99.8 (96.7)	99.5 (98.8)	99.5 (98.9)
Multiplicity[a]	21.6 (20.9)	45.6 (40.6)	13.1 (12.9)	5.5 (5.3)
$I/\sigma I$[a]	9.7 (1.2)	19.0 (1.2)	10.2 (1.5)	5.6 (1.1)
CC(1/2)[1] (%)[a]	99.8 (40.3)	100.0 (44.9)	99.6 (48.8)	99.0 (32.5)
Rpim[2] (%)[a]	4.9 (83.4)	1.7 (80.6)	4.4 (79.8)	8.5 (76.6)
**Refinement**				
Resolution (Å)	42.47–1.95	42.26–1.25	41.86–1.80	45.68–2.20
No. free reflections	1611	2000	1948	2003
R-factor / R-free	20.46 / 22.88	17.12 / 18.95	17.50 / 20.76	22.63 / 26.08
Bond distance (RMS Å)	0.002	0.005	0.008	0.005

Bond angles (RMS °)	0.58	0.84	0.95	0.79
**Structure/Stereochemistry**				
No. atoms: protein	1661	3467	1639	6491
No. atoms: DNA	–	–	–	1220
No. atoms: water	137	236	127	428
Average B-factor: protein	35.44	25.45	32.84	42.99
Average B-factor: DNA	–	–	–	87.48
Average B-factor: water	40.47	34.66	39.99	44.51
Ramachandran plot: favored	99.05%	98.17%	99.04%	97.95%
Ramachandran plot: allowed	0.47%	1.37%	0.96%	1.93%
Ramachandran plot: outliers	0.47%	0.46%	0%	0.12%
Rotamer outliers:	0.56%	0%	1.13%	3.12%
MolProbity[3] score	1.12	1.21	0.98	1.79
Protein Data Bank ID	7TQP	7TQO	7TQN	7TQQ

[a] Highest resolution shell values in parenthesis
[1] (Karplus and Diederichs, 2012)
[2] (Weiss, 2001)
[3] (Chen et al., 2010)
